# Recycling of Technogenic CoCrMo Alloy by Electron Beam Melting

**DOI:** 10.3390/ma15124168

**Published:** 2022-06-12

**Authors:** Katia Vutova, Vladislava Stefanova, Vania Vassileva, Stela Atanasova-Vladimirova

**Affiliations:** 1 Institute of Electronics, Bulgarian Academy of Sciences, 1784 Sofia, Bulgaria; vvvania@abv.bg; 2 Department of Metallurgy of Non-Ferrous Metals and Semiconductors Technologies, University of Chemical Technology and Metallurgy, 1756 Sofia, Bulgaria; vps@uctm.edu; 3 Academician Rostislav Kaishev Institute of Physical Chemistry, Bulgarian Academy of Sciences, 1113 Sofia, Bulgaria; statanasova@ipc.bas.bg

**Keywords:** technogenic Co–Cr–Mo alloy, electron beam recycling, refining process, degree of removal

## Abstract

In the current work, the possibility of the recycling of technogenic CoCrMo material by electron beam melting is investigated. The influence of thermodynamic and kinetic parameters (temperature and melting time) on the behavior of the main components of the alloy (Co, Cr, and Mo) and other elements (Fe, Mn, Si, W, and Nb) present in it, and on the microstructure of the ingots obtained after e-beam processing is studied. The vapor pressure of the alloy is determined taking into account the activities of the main alloy components (Co, Cr, and Mo). The relative volatility of the metal elements present in the alloy was also evaluated. An assessment of the influence of the temperature and the retention time on the degree of elements removal from CoCrMo technogenic material was made. The results obtained show that the highest degree of refining is achieved at 1860 K and a residence time of 20 min. The conducted EDS analysis of the more characteristic phases observed on the SEM images of the samples shows distinct micro-segregation in the matrix composition.

## 1. Introduction

Co-based alloys have been used for more than 80 years as metal biomaterials in dental and surgical prosthetics. In recent years, Co–Cr alloys, additionally alloyed with Mo, W, Ni, and Ti, have become widely used due to their high biological tolerance, low carcinogenicity, excellent mechanical properties and high corrosion resistance [1,2,3,4,5,6]. An improvement in the composition of the alloy can also be achieved by adding some alloying elements such as Si, Nb, and Ir in low concentrations, heat treatments, and developing new processes for production and casting of alloys [3].

Co-based alloys can be classified into two main groups cast—Co–Cr–Mo alloys and hot forged Ni–Co–Cr–Mo alloys. They usually contain 58–69% Co, 26–30% Cr, 5–7% Mo, and other metals such as Ni, Fe, Mn, Si, and W in precisely defined amounts and they are also known as ASTM F75 alloys [7]. Conventional Ni–Co–Cr–Mo alloys are widely used in prosthetics and in the manufacture of the so-called “stents” used for coronary heart disease. They are additionally alloyed with C for greater wear resistance. The main problem with these alloys is still their low ductility and the release of nickel ions during operation.

Cobalt is a metal that determines the basic mechanical properties of the alloy, such as hardness, strength, and toughness [8]. Chromium, whose content is up to 30%, provides biocompatibility and corrosion resistance by forming a protective oxide layer (Cr_2_O_3_) that prevents the diffusion of metal atoms and their contact with oxygen. The molybdenum content in Co–Cr–Mo alloys is usually about 5%. Molybdenum protects the alloy from the action of halogens and their compounds. Together with manganese, it contributes to the fluidity of the alloy. Iron is an inevitable component of the alloy. It improves the machining of the alloy and reduces its electrochemical stability. Silicon, like manganese, contributes to the detoxification and cleansing of the alloy during melting. Carbon protects the alloy from oxidation. However, its content must be strictly controlled (<0.35%) because it tends to form carbides with the metals present in the alloy, thus negatively affecting the basic properties of the alloy such as elasticity and hardness.

There are a number of studies in the literature on the microstructure of Co–Cr–Mo alloys, taking into account the differences in the composition of the samples, the type, and content of the alloying elements, the methods of melting and hardening, the melting temperature, the retention time, etc. [3,6,7,8,9,10,11,12,13,14].

Co–Cr–Mo alloys can be obtained both by conventional casting processing and by other alternative methods such as selective laser and electron beam melting, computer-aided design/computer-aided manufacturing (CAD/CAM) and others [10,11,13,14,15,16,17]. The electron beam melting (EBM) method, which combines the advantages of vacuum and high-energy special electrometallurgy, deserves special attention [18,19,20]. The method allows for the removal of components and ensures the production of pure metals and alloys in the processing of technogenic materials [21,22,23,24].

In this work, the possibility of recycling of technogenic CoCrMo material (waste from the dental technology) by electron beam melting to find technological solutions for refining the alloy is investigated. For this purpose, the influence of thermodynamic and kinetic parameters (treatment temperature and refining time) on the behavior of the main components and the alloying elements (W, Fe, Si, Mn, and Nb), and on the microstructure of the ingots obtained after electron beam melting is studied. Based on thermodynamic analysis and conducted experiments, an assessment of the efficiency of the refining process during EBM was made.

## 2. Material and Methods

The experiments for CoCrMo technogenic material melting were conducted using EBM furnace ELIT-60 (Leybold GmbH, Cologne, Germany) with power 60 kW at the Physical problems of the e-beam technologies laboratory of the Institute of electronics, Bulgarian Academy of Sciences. ELIT-60 is equipped with a melting chamber and one electron gun with an accelerating voltage of 24 kV. The refined metal material solidifies in a water-cooled copper crucible with moving bottom [18,22] and the operation vacuum pressure is 1 × 10^−3^ Pa.

**Figure 1:**
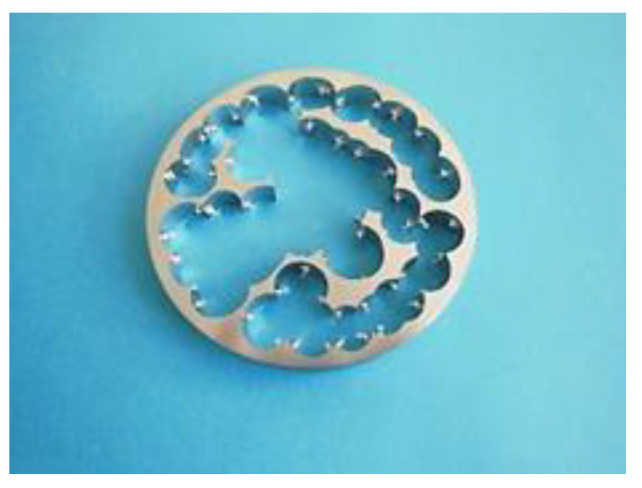
Technogenic CoCrMo material.

The tested material is a technogenic CoCrMo alloy—waste from the dental technology, used in the dental practice for the manufacture of dentures, implants and other products (Figure 1).

Data about the chemical composition of the investigated CoCrMo alloy before EBM is presented in Table 1. The chemical composition of Co–Cr alloy (F75-12) registered in the ASTM standards for biomedical applications [1] is also shown in Table 1.

The table shows that the investigated technogenic material differs from the ASTM F75-12 standard in the chemical composition of the main components and the reduced content of Co and Mo and the high content of the Cr, W, and Si elements require its refining.

A refining of the source material was performed at electron beam power (P_b_) of 2.25 kW (T = 1790 K), 3.75 kW (T = 1830 K), 4.50 kW (T = 1845 K), 4.75 kW (T = 1855 K), and 5.0 kW (T = 1860 K) and refining duration of 10 min, 20 min, and 30 min. For each technological mode the changes in the composition and structure of the material after the e-beam process were controlled. The degree of refining of each of the controlled elements for each technological mode was calculated.

The temperature was determined by an optical pyrometer QP-31 using special correction filters.

The chemical composition of the source material and the specimens after the EBM was determined by emission spectral analysis. The baseline and final concentrations were controlled for both the main components Co, Cr, and Mo as well as for the Fe, Mn, Nb, W, and Si elements.

The preparation of the samples for the metallographic study includes standard procedure, grinding, polishing, and etching. A reagent Glyceregia prepared from 15 mL of HCl, 10 mL glycerol, and 5 mL HNO_3_ [25] was used for etching. The time to manifest the macro and microstructure of the examined samples is ~20 min.

Light microscopy and scanning electron microscopy (SEM) are employed to investigate the macro and microstructure of the ingots (in the centre and periphery) obtained after the EBM.

A light microscope Leica DM2500 (Leica Microsystems GmbH, Wetzlar, Germany) with a digital camera Leica EC3 (Leica Microsystems GmbH, Wetzlar, Germany) was used for the topographic study of the macrostructure after e-beam processing of the specimens. The Leica LAS software (Leica Microsystems GmbH, Wetzlar, Germany) was used for image processing.

The microstructure of CoCrMo alloys was investigated using a Scanning Electron Microscope JEOL 6390 with INCA Oxford EDS detector and an elemental chemical analysis of the composition of the phases observed on the SEM/BEC images was made by Energy Dispersive Spectroscope (EDS) analysis.

## 3. Results and Discussion

### 3.1. Thermodynamic Conditions of Element Volatilization during Electron Beam Melting and Refining

Depending on the thermodynamic conditions of the EBM process and on the type of the removed component, the refining processes could be realized through the following methods: degassing—removal of components with a partial pressure, which is higher than the vapor pressure of the base metal; and distillation—evaporation of more volatile compounds of the metallic components [18].

**Figure 2:**
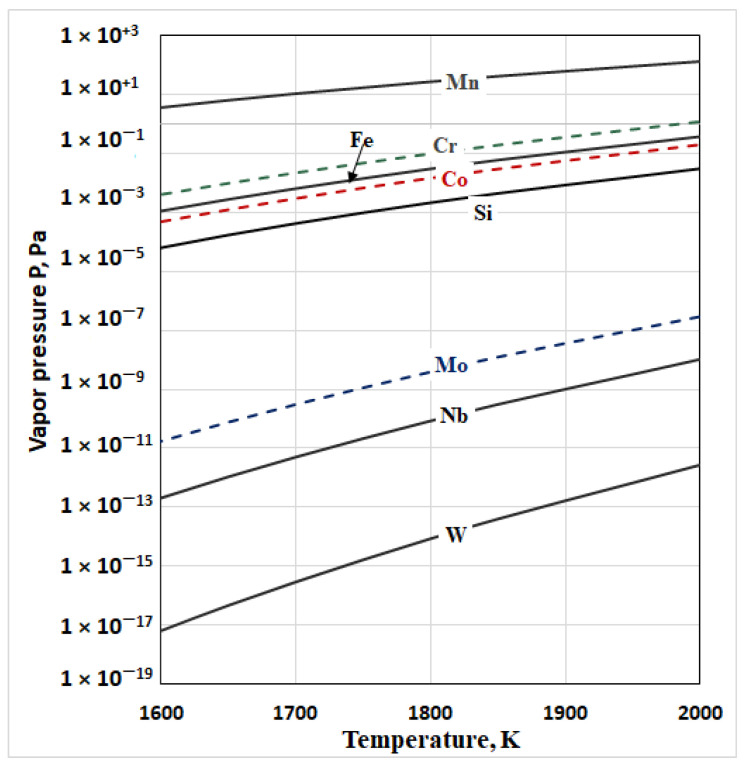
The vapor pressure of the pure elements as a function of the temperature in vacuum.

Figure 2 shows the vapor pressure values of the pure metals (Co, Cr, Mo, Mn, Fe, Si, Nb, and W) present in the studied alloy. They are calculated for a temperature range from 1700 K to 2000 K and an operating pressure in the vacuum chamber of 1 × 10^−3^ Pa. The calculations were made using the professional thermochemical calculation programme HSC Chemistry ver.7.1, module “Reaction Equation” [26].

The figure shows that elements such as W and Nb have a significantly lower vapor pressure than that of the main components of the alloy (Co, Cr, and Mo). Therefore, these elements cannot be removed from the alloy during the EBM. Out of the other controlled elements (Si, Fe, and Mn) only manganese has a vapor pressure higher than that of Co, Cr, and Mo. The iron has a vapor pressure higher than that of Co and Mo and close to that of Cr, while the vapor pressure of silicon is close to that of cobalt. Therefore, in the tested temperature range, these elements can be removed from the reaction surface liquid material/vacuum. Out of the main components of the alloy, chrome has the highest vapor pressure while that of Mo is very low. Therefore, more intense evaporation can be expected only for two (Cr and Co) out of the three elements.

The criterion for assessing the effectiveness of the refining process for multi-component metal systems where the metal or metal alloy is melted in a vacuum is the relative volatility (*α*) [27]. It can be calculated by an equation:

(1) $$\alpha_{i}=\frac{p_{alloy}}{p_{i}}\frac{\sqrt{Mi}}{\sqrt{M_{alloy}}}$$

where *p_alloy_* is the cumulative vapor pressure of the main components of the alloy (Co, Cr, and Mo); and *p_i_*—is the vapor pressure of the other elements present in the alloy (Si, Fe, Mn, W, and Nb). The molecular mass of CoCrMo alloy (*M_alloy_*) is calculated from the expression:

(2) $$M_{alloy}=M_{Co}x_{Co}+M_{Cr}x_{Cr}+M_{Mo}x_{Mo}$$

where *M_Co_*, *M_Cr_*, and *M_Mo_* are the molecular masses of cobalt, chrome, and molybdenum and *x_Co_*, *x_Cr_*, and *x_Mo_* their mols. *M_i_* are the molecular masses of the other elements present in the alloy.

When calculating the vapor pressure (*p_alloy_*) of CoCrMo alloy, it is necessary to take into account the interaction between cobalt, chrome, and molybdenum in the alloy, i.e., their activity. The actual vapor pressure (*p_j(Me)_*) of each of these components is:

(3) $$p_{j\left(Me\right)}=P_{Me}^{o}a_{j\left(Me\right)}$$

where $P_{Me}^{o}$ is the vapor pressure of the pure metals Co, Cr, and Mo, *a_j(Me)_*—the activities of the same components in the alloy.

A number of theoretical and experimental models for determining the activity of components in binary systems are known in the literature [28,29,30]. To determine the Co, Cr and Mo activity in the alloy this study uses the methodology based on two-component diagrams and is presented in [28]. The activity of components in the alloy is calculated using the equation:

(4) $$log(a_{j\left(Me\right)}^{T})=-\frac{(T_{o}-T_{liq})H_{f}}{4574\cdot T\cdot T_{o}}+\frac{T-T_{liq}}{T}logx_{j}$$

where $a_{j\left(Me\right)}^{T}$ is the activity of an alloy element (*j*) at operating temperature *T*; *T_o_*—the melting temperature of an element; *T_liq_*—the temperature of the liquidus surface at a molar portion ($x_{j}$) of the component in the alloy; and *H_f_*—enthalpy of fusion of a given component.

When using Equation (4) or other similar equations, one should keep in mind that these equations are associated with parameters, which are difficult to determine and always entail an error [28]. Nevertheless, the activities calculated using these equations give approximate information about the activity of the components in a more complex metal system.

Table 2 shows the baseline data and the activities of Co, Cr, and Mo in the investigated alloy calculated according to Equation (4). The liquidus surface temperatures were determined based on the binary systems Co–Cr and Co–Mo. The calculations were performed at an operating temperature of 1860 K and a molar composition corresponding to the starting alloy.

To determine the relative volatility (*α_i_*) of the metal components present in the alloy, first the vapor pressure values for pure metals present in the alloy at T = 1860 K were calculated. For this purpose, regression equations were worked out (Table 3) from the graphic dependencies shown in Figure 2. The high correlation factor (R^2^) shows that they can be successfully used to calculate the vapor pressure of pure metals in the temperature range of 1600 K to 2000 K. The integral value of the vapor pressure of the alloy at an operating temperature of 1860 K is also shown in the same table.

**Figure 3:**
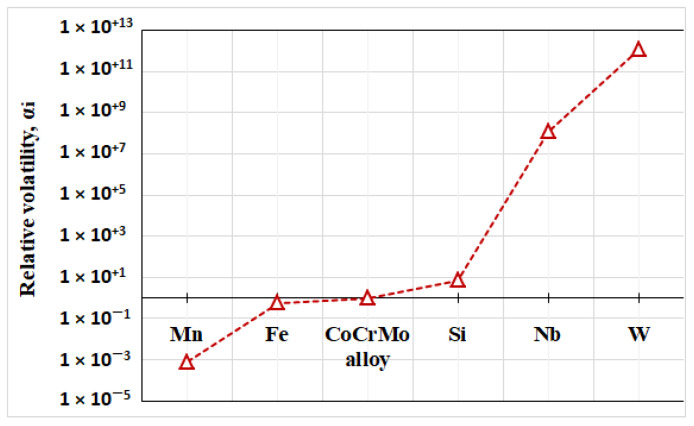
Values of the relative volatility *α_i_* for the metal elements in CoCrMo alloy at 1860 K.

The values of the relative volatility *α_i_* of the metal elements in relation to the CoCrMo alloy at T = 1860 K were calculated using Equation (1) and are shown in Figure 3.

It can be seen that the *α_i_* parameter varies in a wide range from 10^−3^ to 10^1^^2^. For more volatile elements such as Mn and Fe located to the left of the CoCrMo alloy, the values of *α_i_* < 1 and therefore their removal from the alloy is thermodynamically probable. For non-volatile elements (Nb and W) located on the right side of CoCrMo *α_i_* ≫ 1 and therefore their removal is impossible. The removal of Si, whose value is *α_Si_* ~ 1, is possible, but it will be accompanied by significant losses of the alloy mass.

### 3.2. Refining Efficiency during EBMR of Technogenic CoCrMo Alloy

Table 4 shows the chemical composition of ingots after melting in different technological modes (heating temperature and residence time).

The analysis of the results shows that as the temperature and retention time increase, the alloy is enriched in cobalt from 61% to 64.94%. The content of chromium decreases from 31.22% to 28.79%, and that of molybdenum slightly increases—from 4.78% to 5.06%. This can be explained keeping in mind that the vapor pressure of chromium is higher than that of cobalt, while the vapor pressure of molybdenum is much lower than that of Co and Cr (Figure 2).

**Figure 4:**
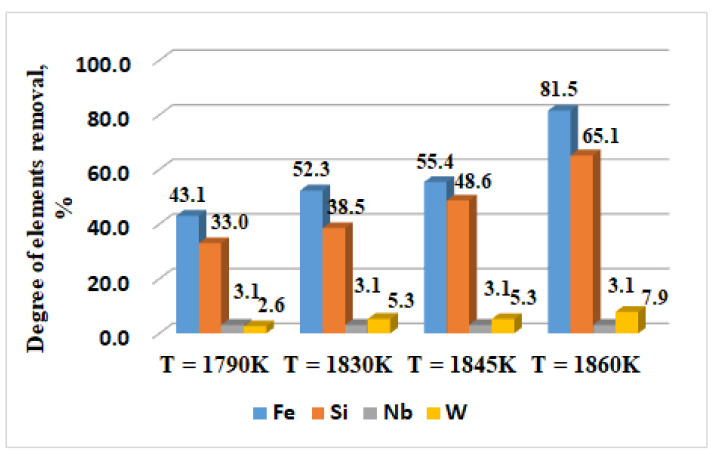
Influence of the melting temperature on the degree of alloying elements removal from the technogenic CoCrMo alloy.

**Figure 5:**
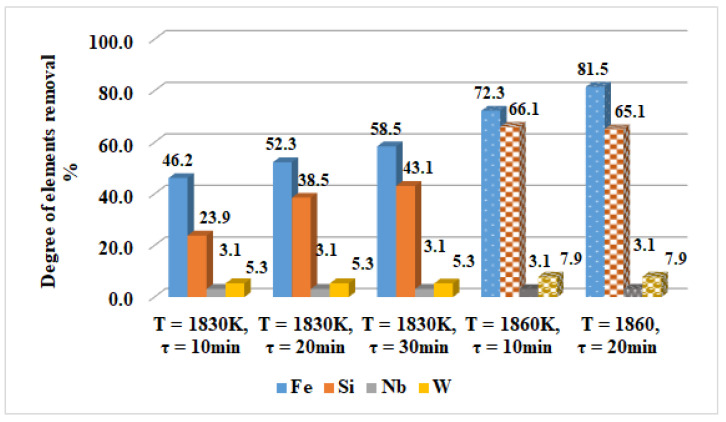
Influence of the melting time on the degree of elements removal at T = 1830 K and T = 1860 K from the technogenic CoCrMo alloy.

For an easier interpretation of the behaviour of elements such as Fe, Mn, Si, Nb, and W, the influence of the temperature (Figure 4) and the retention time of 1830 K and 1860 K (Figure 5) on the degree of removal from CoCrMo alloy are expressed graphically. Mn is not shown in the figures as it is completely removed at 1790 K.

The influence of the temperature in the range from 1790 K to 1860 K at a retention time τ = 20 min on the degree of removal of the elements Fe, Si, Nb, and W is presented in Figure 4. The degree of refining (*βi*) is calculated using the equation:

(5) $$\beta_{\left(i\right)}=\frac{C_{i\left(initial\right)}-C_{i\left(final\right)}}{C_{i\left(initial\right)}}\cdot 100\;\%$$

where $C_{i\left(initial\right)}\;$ and $C_{i\left(final\right)}\;$ are the initial and final content of the i-th element in the alloy, respectively.

The analysis of the results obtained indicates that the Fe and Si removal degree is constantly increasing with the increase in the temperature and at a maximum operating temperature (T = 1860 K, τ = 20 min) reaches 81.5% and 65.1%, respectively (Figure 4). With an extension of the retention time over 20 min, especially at a lower temperature (1830 K), the removal degree of the two elements changes negligibly. At a higher temperature and an extension of the retention time from 10 min to 20 min, the removal rate of iron (*β_Fe_*) is increased by about 9% and hardly influences that of silicon (Figure 5). With regard to the more non-volatile elements such as Nb and W, the degree of removal is average ~3% for niobium and ~6% for W. Therefore, these elements cannot be refined by evaporation even at the highest temperature and extension of the melting time.

The conclusions drawn on the basis of the experiments carried out fully confirm the conclusions made on the basis of the thermodynamic analysis of the behaviour of the alloying elements present in the CoCrMo alloy.

The results obtained show that the highest degree of refining is achieved at T = 1860 K and a retention time of 20 min. The chemical composition of the alloy complies with the standard ASTM F75-12 for biomedical applications with the exception of tungsten, the concentration of which is slightly higher than the permissible amount. There are no requirements for the niobium content in the standard. Both alloying elements (W and Nb) improve the composition and structure of the alloy [3]. Tungsten stabilizes the hexagonal close-packed (HCP) structure and Nb stabilizes the face-centered cubic (FCC) structure [1].

### 3.3. Microstructures of CoCrMo Alloy after EBM

The main components in the studied CoCrMo alloy are Co (~61%), chromium (~31%), and molybdenum (~5%) and the phase changes that take place in the alloy during cooling after the EBM may be described with the Co–Cr–Mo phase diagram or the Co–Cr, Co–Mo, and Cr–Mo two-component diagrams.

**Figure 6:**
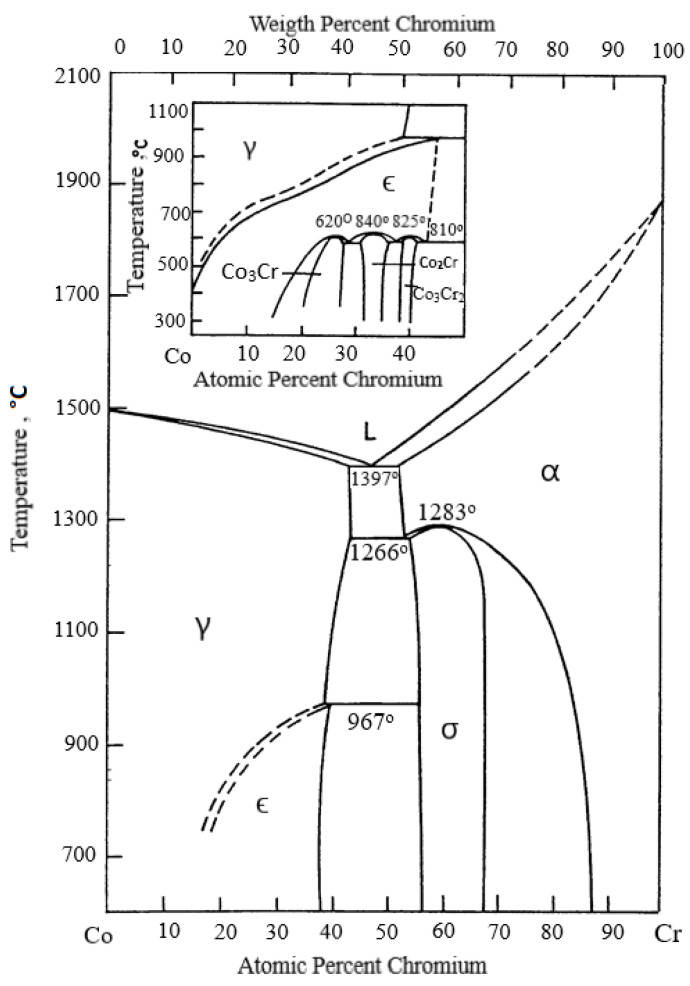
Two-component phase equilibrium diagram Co–Cr [31,32,33].

Figure 6 shows the Co–Cr phase equilibrium diagram. At a Cr content of ~31%, the solidification of the alloy starts approximately at a temperature of 1670 K, avoiding the eutectic transition [31,32,33]. The high temperature γ-phase with a face-centered cubic (FCC) grid, which is stable up to ~1223 K, solidifies directly from the liquid. After that the martensitic transformation of the γ-phase starts up to a low temperature hexagonal close-packed (HCP) ε-phase. There is a eutectoid decomposition of the ε-phase → Co_3_Cr + Co_2_Cr at lower temperatures. At a higher concentration of Cr, the decomposition takes place along the reaction: ε-phase → Co_2_Cr + Co_3_Cr_2_.

The Co–Mo diagram analysis shows that the Mo melting temperature is much higher than that of Co (T_m,Mo_ = 2896 K, T_m,Co_ = 1768 K) and the eutectic mixture was obtained at a temperature of 1608 K and approximately 40 mass% of Mo.

When examining Cr–Mo alloys it was found that chromium and molybdenum formed a spinoidal mixture. In the presence of Si, the molybdenum forms intermetallic compounds of the type of Mo_5_Si_3_ and Mo_3_Si. At lower temperatures and a higher chromium content, σ-phase formation is observed. This phase is an intermetallic Co compound with Cr with a composition corresponding approximately to Co_2_Cr_3_ and in the presence of molybdenum—Co_x_Cr_y_Mo_z_.

The impact of other alloying elements on the transformation temperature from the HCP to the FCC phase is summarized in [34] and elements such as Fe, Mn, Ni, Nb, and C reduce the temperature of the transformation from HCP to FCC, i.e., they are stabilisers of the FCC phase. Metals such as Cr, Mo, W, and Si increase the temperature of the transformation from HCP to FCC, therefore they are stabilisers for the HCP phase. These transformations are closely related to the alloy microstructure and hence, its mechanical and chemical properties.

**Figure 7:**
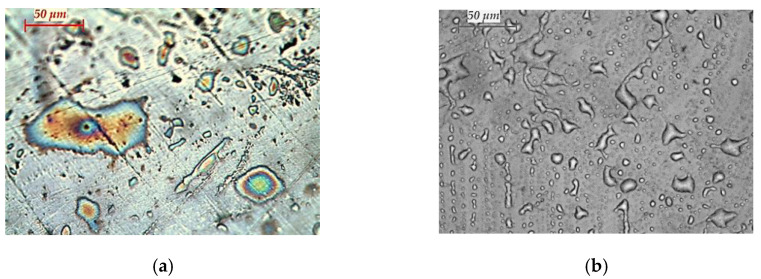

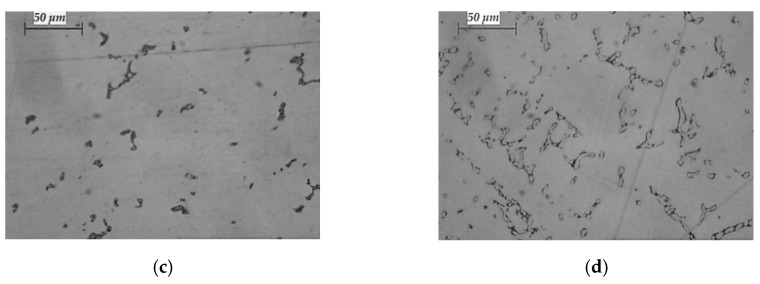
Optical micrographs of: (**a**) initial CoCrMo alloy; and ingots obtained after EBM: (**b**) T = 1790 K; (**c**) T = 1845 K; and (**d**) T = 1860 K; τ = 20 min (400× magnification).

Figure 7 shows a microstructure of samples of CoCrMo alloy before and after EBM at temperatures of 1790 K, 1845 K and 1860 K and a retention time of 20 min.

The analysis of the microstructure of the starting sample indicates that it is highly oxidised and with a lot of defects. After an electron beam melting of the alloy at a temperature of 1790 K, a large number of intermetallic compounds situated on a Co matrix are observed on the surface. This can be explained by keeping in mind the low removal degree of the elements at this temperature (Figure 4).

With an increase in the temperature to 1860 K, the formation of a dendritic γ-phase, rich in cobalt, is observed. In this case, the degree of iron refining is very high (>80%), and that of Si is ~65%. W and Nb remain in the sample and it is only Nb that is a stabiliser of the γ-phase.

**Figure 8:**
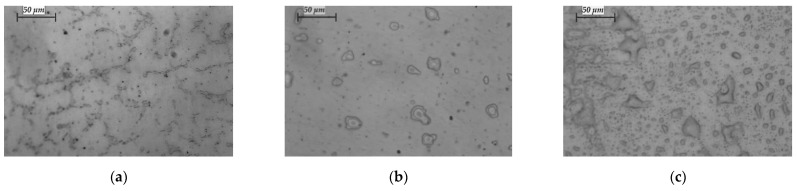
Microstructures of CoCrMo specimens manufactured at T = 1830 K for different refining time: (**a**) τ = 10 min; (**b**) τ = 20 min; and (**c**) τ = 30 min (400× magnification).

Microstructures of samples obtained at T = 1830 K and retention time τ = 10, 20 and 30 min are shown in Figure 8.

It is obvious that prolongation of the residence time results in the formation of a large number of intermetallic melts in the HCP phase. The transformation of the FCC structure to HCP is a very slow process and depends on both the melting parameters and the type of elements present in the alloy. The low refining degree of W and Si (Mn is completely removed at a temperature of 1790 K) from the CoCrMo alloy at T = 1830 K leads to the stabilisation of the low-temperature ε-phase [1]. This in turn leads to a decrease in the mechanical properties of the alloy and an increase in its corrosion resistance.

On the basis of the metallographic analysis using light microscopy, only a qualitative microstructure assessment of the samples can be provided. To obtain more accurate information on the influence of the EBM technological parameters on the microstructure of the ingots obtained, a microscopic study was performed using SEM and an elemental chemical analysis of the phases observed on the SEM/BEC images was made by EDS analysis. For each more characteristic phase (such as the colour and shape), at least 25 spectra are taken and the average chemical composition of the phase is calculated; they are labelled as “dark”, “grey”, and “light” phases, respectively.

**Figure 9:**
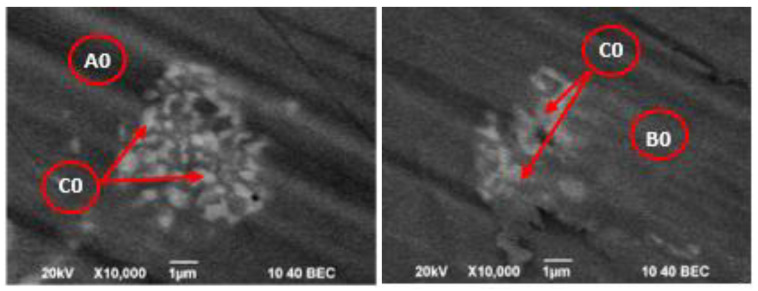
More characteristic phases observed on the SEM/BEC images of the CoCrMo material before e-beam melting (sample Co-0).

Table 5 shows the chemical composition of the more characteristic phases observed on the SEM/BEC images of the primary CoCrMo alloy (Figure 9).

The EDS analysis shows that the alloy’s matrix is mainly based on the Co_1.8_Cr phase, close in composition to the Co_2_Cr phase (Figure 6), which is obtained after the decomposition of an ε-phase at a temperature <973 K [31,32,33]. The differences between the “dark” and “grey” phases are mainly due to the different content of Mo (from 7.19 to 7.46 at%) and Nb (from 0 to 0.85 at%). The content of the other elements in these phases is <1 at%.

The “light” phase observed on the SEM photos is a molybdenum-rich phase (Co_0.8_Cr_0.5_Mo_0.2_). The Nb and Si content in this phase is ~8 at% and ~2 at%, respectively.

Regardless of the large number of analyses, two elements were not identified—Mn and W. This can be explained by their low content in the studied alloy (Table 1).

**Figure 10:**
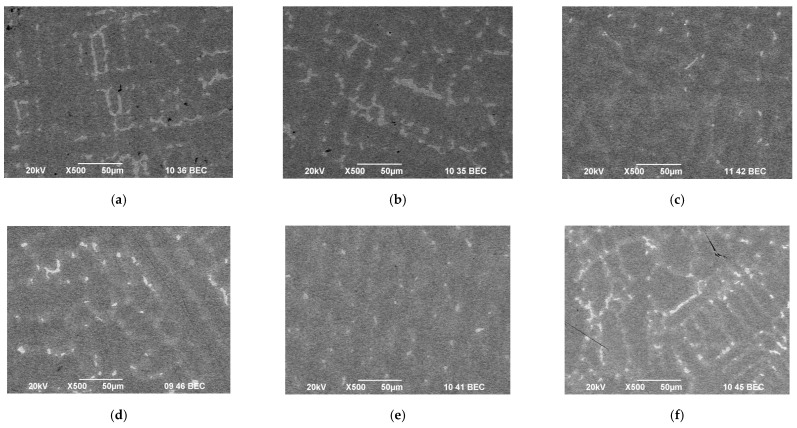
Microstructures of CoCrMo samples produced/manufactured at different process conditions: (**a**,**b**) T = 1830 K, τ = 20 min; (**c**,**d**) T = 1830 K, τ = 30 min; (**e**,**f**) T = 1860 K, τ = 20 min; (**a**,**c**,**e**)—microstructure on the periphery of the specimen; (**b**,**d**,**f**)—microstructure in the central part of the sample.

**Figure 11:**
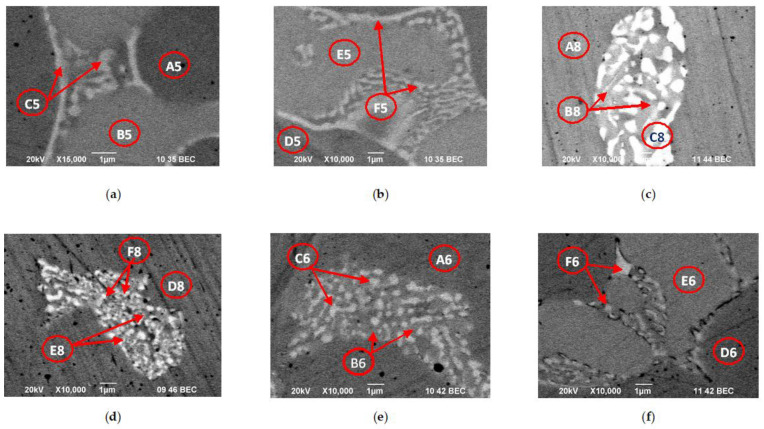
More characteristic phases observed in the CoCrMo specimens after EBM at different modes: (**a**,**b**) T = 1830 K, τ = 20 min; (**c**,**d**) T = 1830 K, τ = 30 min; (**e**,**f**) T = 1860 K, τ = 20 min; (**a**,**c**,**e**)—phases formed on the periphery of the sample; (**b**,**d**,**f**)—phases formed in the central part of the sample.

Figure 10 and Figure 11 show SEM/BEC images of the microstructure and the more characteristic intermetallic phases formed in the CoCrMo alloy after EBM at temperatures of 1830 K and 1860 K and retention time τ = 20 min. At a temperature of 1830 K, an experiment with a residence time of 30 min was performed (sample Co-08). The calculated values of the average chemical compositions of the phases observed on the SEM/BEC photographs are given in Table 6.

Figure 10 shows that the solidification process of CoCrMo alloy is accompanied by the formation of a dendritic structure and the formation of characteristic intermetallic melts. As the temperature increases, the solidification rate decreases, leading to the formation of the HCP structure. This process is more pronounced in the centre of the samples and shows that the process proceeds at the solid/liquid interface in the direction of direct heat removal.

**Figure 12:**
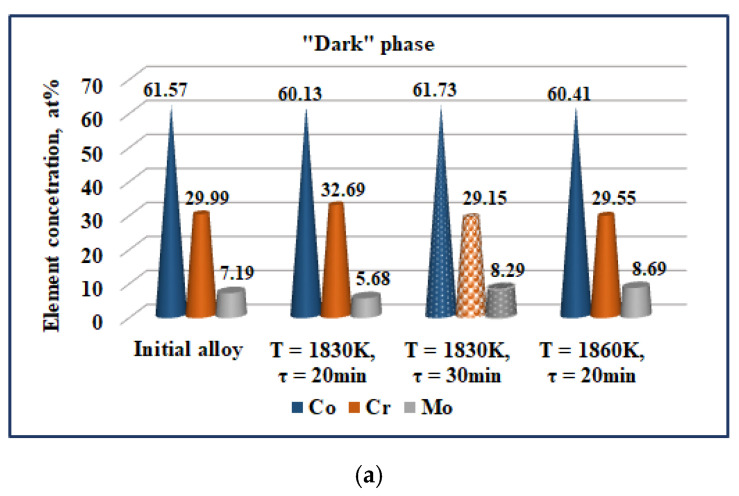

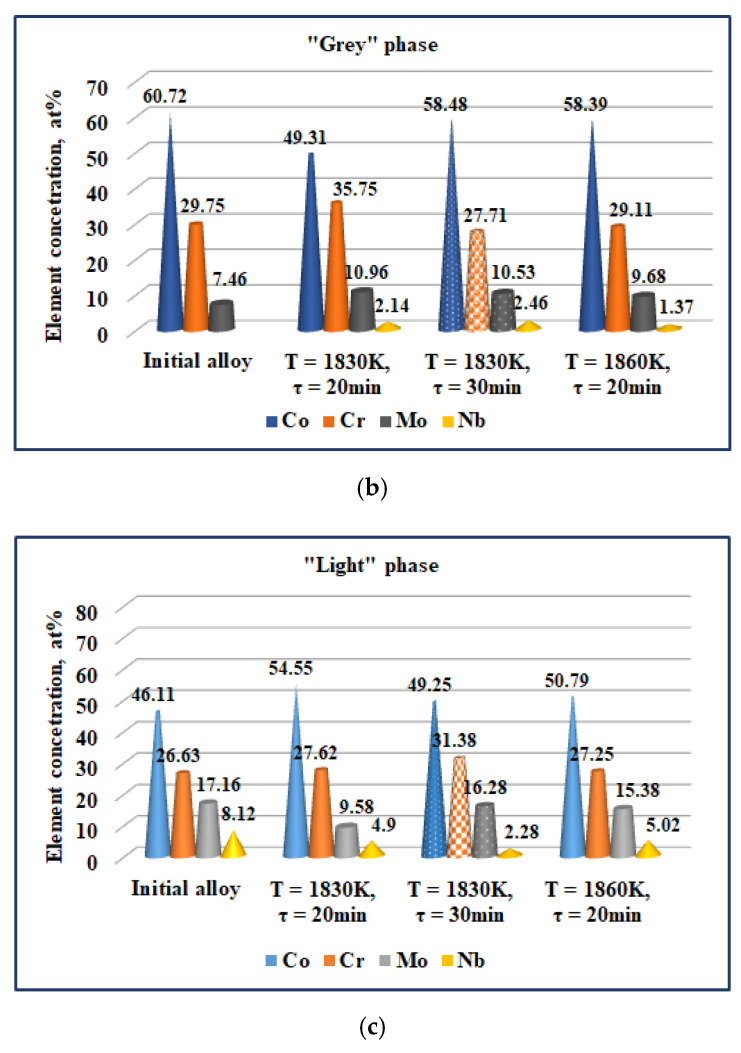
Influence of the temperature and melting time on the composition of the phases observed on the SEM pictures: (**a**) “dark” phase; (**b**) “grey” phase; and (**c**) “light” phase.

The EDS microanalysis of the periphery and the centre of the samples (Table 6) shows non-essential differences in their chemical composition and therefore only the phases identified in the centre of the samples will be discussed. The influence of the temperature and the retention time on the chemical composition of the phases observed on the SEM images is represented in Figure 12.

The EDS analysis shows a distinct micro-segregation in the cobalt matrix composition. At a temperature higher than 1830 K in the inter-dendrite zone (“dark”), a Co_1.8_Cr(Mo,Si) phase is formed. It is close in composition to the Co_2_Cr phase, which is obtained after the decomposition of the ε-phase (Figure 6). The Co content of this phase is ~60 at% and does not change with an increase in temperature, while the Cr content decreases from ~33 at% to approximately 29 at% (Figure 12a). By increasing the temperature, the melt is enriched in Mo by ~3 at%. W is not present in this phase and the Nb content is less than 0.1 at%.

The chemical microanalysis of the “grey” phase shows a lower Co content and higher Mo content compared to the “dark” phase (Figure 12b). Unlike the “dark” phase, the Nb content is ~2 at%.

Out of the other elements present in the cobalt matrix, with the temperature increase, the Si content decreases both in the “dark” phase (from 1.5 to 0.79 at%) and in the “grey” phase (from 1.84 to 0.87 at%).

Chromium, molybdenum, and silicon diffuse to the inter-dendrite zones [35]. This in turn leads to an increase in the reflex’s intensity. In our study it was observed that Nb also diffuses to the inter-dendrite zone of the material.

The chemical composition of the “light” phases shows that they are intermetallic compounds of the type Co_x_Cr_y_Mo_z_(Si,Nb). By increasing the temperature, the melt is enriched in Mo (from 9.58 to 15.38 at%) and Nb, while the Si content decreases (from 3.35 to 1.15 at%). In the studied temperature interval, the Cr content hardly changes, and it is ~27 at%, while the Co decreases slightly from 54 to 51 at%.

The EDS analysis of the “dark” phase shows that an extension of the retention time from 20 to 30 min (samples Co-05 and Co-08) results in a decrease in the Cr content by 3.5 at%, enriching in Mo from ~5.7 to ~8.3 at%. The change in Co content is negligible.

Unlike the “dark” phase, the Co content in the “grey” phase is significantly increasing (from ~49 to ~58.5 at%) with the extension of time, while that of Cr decreases from 35.7 to 27.7 at%. The content of Mo and Nb remains almost constant, and it is ~10 at% (Mo) and ~2 at% (Nb), respectively.

By extending the retention time, the chemical composition of the “light” phase is enriched in Cr (from ~27.6 to ~31 at%) and Mo (from 9.6 to 16 at%). The Nb and Si content in these melts decreases from ~4.9 to ~2.3 at% (Nb) and from ~3.3 to ~0.8 at% (Si), respectively.

## 4. Conclusions

This paper studies the possibility of refining a technogenic CoCrMo alloy using EBM. Based on the thermodynamic analysis, the vapor pressure of the major components of the alloy (Co, Cr, and Mo) and the other elements (Fe, Mn, Si, W, and Nb) present in it was calculated. The integral value of the vapor pressure of the alloy, taking into account the activities of the main alloy components and the relative volatility of the metal elements in the alloy were assessed. An experimental study of the influence of kinetic parameters (temperature and refining time) on the change in the chemical composition of the alloy and the microstructure of the ingots was carried out. The results obtained can be summarised as follows:

- Within the studied temperature range (1600–2000 K), it is most likely to have evaporation of Mn and Fe. Tungsten and niobium have significantly lower vapor pressures than Co, Cr, and Mo and cannot be removed from the alloy. Si has a vapor pressure close to that of Co and its evaporation is thermodynamically probable, but it will be difficult. Out of the main components of the alloy, the probability of evaporation of Cr is the highest.
- It has been found that the removal rate of Fe and Si increases with the increase in the temperature and with an extension of the retention time. It is shown that Mn is completely removed at the lowest operating temperature tested (1790 K) and the degree of removal of non-volatile elements (Nb and W) remains low. The highest degree of refining was achieved at 1860 K and a residence time of 20 min.
- The EDS analysis of the more characteristic phases observed on the SEM images of the samples shows a distinct micro-segregation in the matrix composition. At a temperature higher than 1830 K in the inter-dendrite “dark” zone, a Co_1.8_Cr(Mo,Si) phase is formed.
- It has been found that the “light” phases are intermetallic compounds of the type Co_x_Cr_y_Mo_z_(Si,Nb), which are enriched in Mo and Nb as the temperature increases.

The study has shown the possibility of recycling through EBM of CoCrMo technogenic material for the needs of biomedical practice.

## Figures and Tables

**Table 1:** Chemical composition (mass%) of the investigated technogenic CoCrMo material and Co-Cr alloy (F75-12) according to the ASTM standards.

Sample	Co	Cr	Mo	Ni	Fe	C	Mn	Nb	W	Si	Others
Initial material	61.0	31.22	4.78	0.0	0.65	0.0	0.43	0.32	0.38	1.09	0.13
ASTM F75-12	balance	27–30	5–7	<0.5	<0.75	<0.35	<1.0	-	<0.2	<1.0	<0.49 ^1^

^1^ P < 0.02, S < 0.01, N < 0.25, Al < 0.1, Ti < 0.1, B < 0.01.

**Table 2:** Starting data and activities of Co, Cr, and Mo for initial CoCrMo alloy at T = 1860 K.

Element	x_j_	T_0_, K	H_f_, kJ/mol	T_liq_, K	T, K	$\mathrm{Activity},\;a_{j\left(Me\right)}^{T}$
Co	0.614	1768	16,190	1675	1860	0.804
Cr	0.356	2180	16,900	1688	1860	0.446
Mo	0.030	2896	32,000	1765	1860	0.082

**Table 3:** Regression equations and vapor pressure of the metal elements and alloy at an operating temperature of 1860 K.

No	Equation	R^2^	$P_{\left(Me\right)}^{o}$	$p_{j\left(Me\right)}$
1	$p_{Mn}=2E-6e^{0.009T}$	0.9951	3.72 × 10^1^	-
2	$p_{Fe}=1E-13e^{0.0145T}$	0.9941	5.15 × 10^−2^	-
3	$p_{Si}=1E-15e^{0.0154T}$	0.9935	2.74 × 10^−1^	-
4	$p_{Nb}=4E-32e^{0.0271T}$	0.9958	3.10 × 10^−10^	-
5	$p_{W}=3E-40e^{0.0324T}$	0.9959	4.433 × 10^−14^	-
	$p_{Co}=2E-14e^{0.015T}$	0.9943	2.61 × 10^−2^	2.1 × 10^−2^
	$p_{Cr}=6E-13e^{0.0142T}$	0.9954	1.77 × 10^−2^	7.89 × 10^−3^
	$p_{Mo}=3E-28e^{0.0243T}$	0.9958	7.28 × 10^−9^	5.97 × 10^−10^
6	$P_{alloy}=p_{i\left(Co\right)}+p_{i\left(Cr\right)}+p_{i\left(Mo\right)}$ = 2.89 × 10^−2^			

**Table 4:** Process parameters and chemical compositions (mass%) of the specimens before and after electron beam melting and refining.

Sample	Parameter		Concentration of Basic Elements			Concentration of Other Elements					
	T, K	τ, min	Co	Cr	Mo	Fe	Mn	Nb	W	Si	Others
Co-0	Initial alloy		61	31.22	4.78	0.65	0.43	0.32	0.38	1.09	0.13
Co-07	1790	20	62.11	31.01	4.96	0.37	0.0	0.31	0.37	0.73	0.14
Co-04	1830	10	62.60	30.51	4.91	0.35	0.0	0.31	0.36	0.83	0.13
Co-05	1830	20	62.92	30.41	4.90	0.31	0.0	0.31	0.36	0.67	0.12
Co-08	1830	30	63.54	29.79	4.99	0.27	0.0	0.31	0.36	0.62	0.12
Co-02	1845	20	63.99	29.35	5.05	0.29	0.0	0.31	0.36	0.56	0.09
Co-03	1860	10	64.14	29.81	4.80	0.18	0.0	0.31	0.35	0.37	0.04
Co-06	1860	20	64.94	28.79	5.06	0.12	0.0	0.31	0.35	0.38	0.05

**Table 5:** EDS microanalysis of more characteristic phases observed in the initial CoCrMo alloy.

Sample	Area	Chemical Composition, at%							Phase
		Co	Cr	Mo	Fe	Si	Nb	W	
Co-0	A0 (dark)	61.57	29.99	7.19	0.42	0.83	0	0	Co_1.8_Cr(Mo, Fe, Si)
	B0 (grey)	60.72	29.75	7.46	0.47	0.75	0.85	0	Co_1.8_Cr(Mo, Fe, Si, Nb)
	C0 (light)	46.11	26.63	17.16	0	1.98	8.12	0	Co_0.8_Cr_0.5_Mo_0.2_(Si, Nb)

**Table 6:** Microanalysis of more characteristic phases observed at the periphery and in the center of CoCrMo ingots obtained after e-beam melting.

Sample		Area	Chemical Composition, at%						
			Co	Cr	Mo	Fe	Si	Nb	W
Co-05 T = 1830 K τ = 20 min	Rim	A5 (dark)	59.73	33.55	4.87	0.23	1.62	0	0
		B5 (grey)	48.30	36.83	11.06	0	2.06	1.75	0
		C5 (light)	53.45	28.67	9.78	0.11	3.25	4.74	0
	Core	D5 (dark)	60.13	32.69	5.68	0	1.50	0	0
		E5 (grey)	49.31	35.75	10.96	0	1.84	2.14	0
		F5 (light)	54.55	27.62	9.58	0	3.35	4.90	0
Co-08 T = 1830 K τ = 30 min	Rim	A8 (dark)	61.24	32.41	4.66	0.17	1.52	0	0
		B8 (grey)	50.30	31.81	11.03	0.52	2.85	3.49	0
		C8 (light)	50.65	32.07	11.23	0	2.60	3.45	0
	Core	D8 (dark)	61.73	29.15	8.29	0	0.74	0.09	0
		E8 (grey)	58.48	27.71	10.53	0	0.82	2.46	0
		F8 (light)	49.25	31.38	16.28	0	0.81	2.28	0
Co-06 T = 1860 K τ = 20 min	Rim	A6 (dark)	61.19	29.56	8.28	0.38	0.59	0	0
		B6 (grey)	59.35	29.02	8.89	0.45	0.72	1.57	0
		C6 (light)	54.23	23.85	13.68	0	1.59	6.65	0
	Core	D6 (dark)	60.41	29.55	8.69	0.46	0.79	0.10	0
		E6 (grey)	58.39	29.11	9.68	0.58	0.87	1.37	0
		F6 (light)	50.79	27.25	15.38	0.41	1.15	5.02	0
